# Research advances in the degradation of aflatoxin by lactic acid bacteria

**DOI:** 10.1590/1678-9199-JVATITD-2023-0029

**Published:** 2023-10-23

**Authors:** Yuxi Wang, Lishi Jiang, Ying Zhang, Ran Ran, Xiao Meng, Shukun Liu

**Affiliations:** 1 Institute of Public Health, Chengdu University of Traditional Chinese Medicine, Chengdu, China.; 2 School of Light Industry and Materials, Chengdu Textile College, Chengdu, China.

**Keywords:** Aflatoxins, applications, biodegradation, lactic acid bacteria, mechanism

## Abstract

Aflatoxins are toxic secondary metabolites that often contaminate food and animal feed, causing huge economic losses and serious health hazards. Aflatoxin contamination has become a major concern worldwide. Biological methods have been used to reduce aflatoxins in food and feed by inhibiting toxin production and detoxification. Among biological methods, lactic acid bacteria are of significant interest because of their safety, efficiency, and environmental friendliness. This study aimed to review the mechanisms by which lactic acid bacteria degrade aflatoxins and the factors that influence their degradation efficiency, including the action of the lactic acid bacteria themselves (cell wall adsorption) and the antifungal metabolites produced by the lactic acid bacteria. The current applications of lactic acid bacteria to food and feed were also reviewed. This comprehensive analysis provided insight into the binding mechanisms between lactic acid bacteria and aflatoxins, facilitating the practical applications of lactic acid bacteria to food and agriculture.

## Background

Fungal contamination of food and feed commodities has become common because of the ubiquitous nature of many fungi and their capability to grow under various environmental conditions. Mycotoxins are secondary metabolites produced by fungi during their growth process, which not only significantly harm the health of humans and animals but also cause substantial economic losses. The prevalence of mycotoxins in food crops has been reported to be 60%-80%, with annual economic losses of over US$ 932 million globally for agricultural products contaminated with mycotoxins [1]. The level of toxic mycotoxins entering the food chain has increased due to climate change worldwide and improper storage in developing countries [2]. Consumption of food and feed contaminated with fungi and their mycotoxins has carcinogenic, teratogenic, mutagenic, and immunosuppressive effects on humans and animals [3].

The fungi that contaminate food and feed are classified into *Aspergillus*, *Penicillium*, *Fusarium*, and so forth. High temperatures and humid environmental conditions promote the growth of fungi and the production of toxins, which mainly include aflatoxins, fumonisins, ochratoxins, zearalenone, and so forth [4]. The most widely known mycotoxins are aflatoxins (AFs), which are the most toxic mycotoxins creating the most problems. Ingestion of AF-contaminated food may lead to various complications, such as hepatotoxicity, teratogenicity, and immunotoxicity, posing a serious threat to human and animal health [5]. Aflatoxins have been shown to have serious liver carcinogenicity. Aflatoxin B1 (AFB1) is a major cause of hepatic cell carcinoma (HCC) worldwide. Chronic liver injury in humans is caused by frequent exposure to AFs, and HCC is easy to develop if not monitored for a long time [6].

As a result, several physical and chemical methods have been used to remove mycotoxins from food and feed, but for a variety of reasons they have not met the needs of the population. Nowadays, biodegradation methods are being extensively investigated to solve the problem of mycotoxin contamination in our daily lives. This study aimed to review the latest research progress on the degradation of AFs using lactic acid bacteria (LAB). 

**Figure 1.**
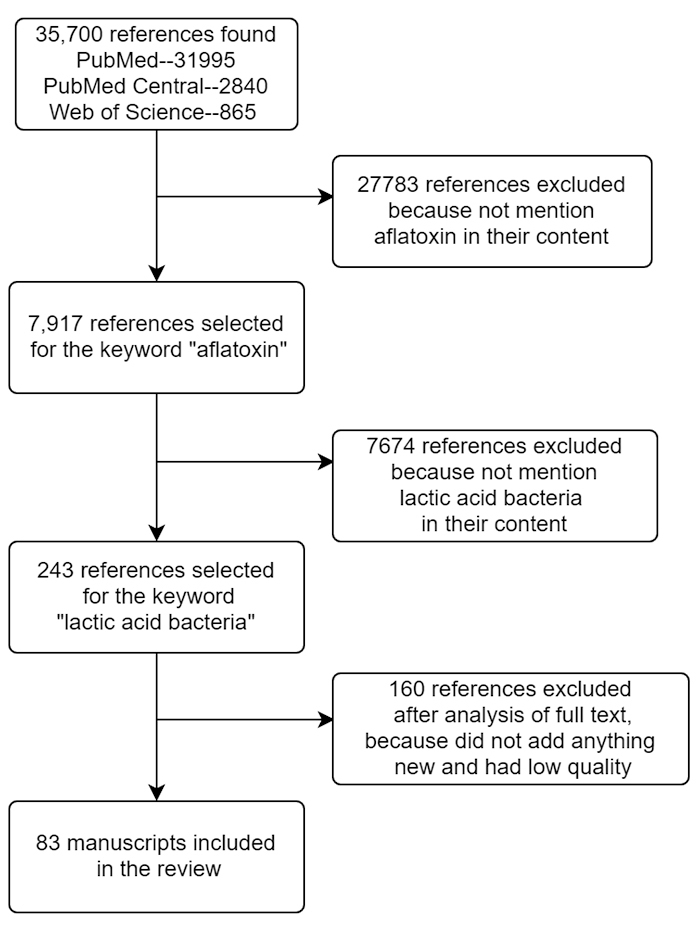
Article selection flow chart.

Search engines PubMed, PubMed Central, and Web of Science were used for this review. Keywords searched include “mycotoxins” or “aflatoxin”, “lactic acid bacteria” or “*Lactiplantibacillus plantarum*”, and “degradation”, “adsorption”, and “biointegration”. First, articles that do not deal with aflatoxin at all were excluded. From these, articles in which LAB (including *Lactiplantibacillus plantarum, Lactobacillus rhamnosus*, *Lactobacillus kefiri, Lactobacillus reuteri and so on*) play a role were then screened. Articles that did not bring anything new to the table were excluded after a complete reading. Figure 1 provides a better description of the literature selection as an article selection flowchart.

### Aflatoxin

*Definition and sources of aflatoxins*

Aflatoxin is a highly toxic, low-molecular-weight secondary metabolite produced by fungi such as *Aspergillus flavus*, *A. parasiticus*, and *A. specific*, which are highly toxic, mutagenic, and carcinogenic [7]. More than 20 AFs have been identified to date, with aflatoxins B1 (AFB1), B2 (AFB2), G1 (AFG1), G2 (AFG2), and M1 (AFM1) being the most common, and AFB1 being the most toxic and carcinogenic [8]. Aflatoxins are most likely to be found in food and feed in hot and humid regions. They are found in soil, plants, animals, and various nuts, and are particularly likely to contaminate grains such as peanuts, maize, rice, soybeans, wheat, and oil seeds [9].

*Dangers of aflatoxins*

Infection caused by aflatoxins can cause economic losses and health risks. The economic losses are reflected in reduced yields, loss of nutritional value, and reduced market value of agricultural products. Commodities such as maize, peanuts, pistachios, almonds, walnuts, and cottonseed are highly susceptible to AFs contamination [10]. Aflatoxins are associated with acute toxicity and chronic carcinogenicity in human and animal populations [11]. Symptoms of acute AFs poisoning include vomiting, abdominal pain, edema, hemorrhagic necrosis of the liver, and severe drowsiness [10]. Acute poisoning, although generally rare in developed countries, is common in some developing countries (especially in regions of Africa), while chronic carcinogenicity is a global problem [12]. Aflatoxin B1 is classified as a Group 1 carcinogen in humans by the International Agency for Research on Cancer (IARC) [13]. Aflatoxins specifically target the liver organ, where bioactivated AFB1 can bind to DNA and cause a base G-T reversal. The human *p53* gene is the main target of the AFB1-DNA adduct. Therefore, the carcinogenicity of HCC caused by AFs is mainly due to oxidative damage to DNA [14]. Additionally, AFs are teratogenic, mutagenic, and immunosuppressive.

*Degradation of aflatoxins*

Several strategies are already in place to prevent or eliminate AFs contamination to restore the safety and edibility of food and feed. Degradation methods are mainly classified into physical, chemical, and biological methods. The physical methods for removing AFB1 from food most commonly use microwave and gamma-ray heating. Patil et al. [15] used low-power microwave heating (360, 480, and 600 W) to reduce the AFB1 content by 59%-67% in peanuts. Studies have also reported using γ-rays (such as ^60^Co) to irradiate foods on several different food matrices, including peanuts, cereals, and animal feed, employing ionizing radiations ranging from 3 to 10 kGy [16]. By exposing solutions of water, buffers, and acids to a plasma discharge, cold plasma-induced ions (H^+^, H_3_O^+^, O^+^, H^-^, O^-^, and OH^-^), molecules (N_2_, O_2_, O_3_, and H_2_O_2_), and reactive radicals (O^-^, H^-^, OH^-^, and NO^-^) are formed, which collectively are called reactive chemicals. The production of these reactive substances has led researchers to use cold plasma to degrade various toxins, including AFs [17]. Porto et al. found a 57% reduction in AFs levels in maize pellets following ozonation treatment [18]. Many physical and chemical methods have been shown to reduce AFs levels. However, these methods have several negative effects, including the possibility of producing other harmful substances, the possibility of altering food properties, and the high cost of use, thereby failing to meet the FAO (Food and Agriculture Organization of the United Nations) requirements of reducing AFs without residual toxicity, ensuring nutritional value, and not altering food or feed properties [19]. 

Therefore, alternative, safe, and cost-effective strategies must be explored to prevent or reduce mycotoxin contamination in food and feed. At present, biological detoxification methods are widely studied for their safety, environmental friendliness, and high efficiency. Of these, LAB are the most promising fungal antagonists for use in food, as they have been used extensively in traditional food fermentation since ancient times. In terms of the biodegradation of AFs, approximately 17% of the studies were related to the degradation of AFs using *Lactobacillus* [20]. *Lactiplantibacillus plantarum (L. plantarum)* also belongs to the genus *Lactobacillus*.

### Lactic acid bacteria and their mechanism of action

*Potential of lactic acid bacteria to inhibit fungi*

Many different sources of LAB have been shown to have antifungal activity and the potential for biopreservation (Table 1). Notably, *L. plantarum* is the best-known LAB species in terms of fungal inhibitory activity. Ahlberg et al. [21] classified species belonging to the genera *Lactococcus*, *Lactobacillus*, *Pediococcus*, and *Leuconostoc* as capable of inhibiting the growth of toxin-producing fungi or inhibiting mycotoxin species. Among the 23 strains of LAB isolated from traditional Egyptian fermented milk, strain RM1 had the highest antifungal capacity, with complete inhibition of both AFB1 and ochratoxin A production at a free supernatant concentration of 15 mg/mL of strain RM1 [22]. Russo et al. [23] used the plate overlap method to screen the antifungal activity of *L. plantarum* 88 strains against *A. flavus*, *A. niger*, *Penicillium dilatancy*, *P. roqueforti*, *Fusarium*, and *Cladosporium*, reporting significant phenotypic heterogeneity among their antifungal activity traits. Similarly, Nazareth et al. [24] reported that the cell-free supernatant (CFS) of *L. plantarum* CECT 749 showed good inhibition of *A. flavus* and *Fusarium flavus* infesting maize seeds, reducing mycotoxin production by 73.7%-99.7%. Numerous studies have identified different strains of LAB, all with potent antifungal activity.

**Figure 2.**
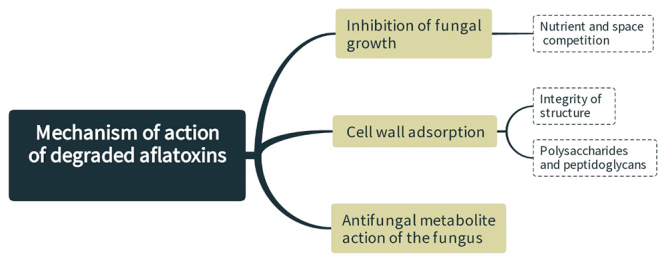
Mechanism of action of lactic acid bacteria in the degradation of aflatoxins. The inhibition may be the effect of these mechanisms acting together.

However, the effectiveness of different LAB in controlling mycotoxin contamination varies from strong to weak, suggesting that not all strains of the species have the same potential to avoid fungal spoilage. The reason is the differences in the mechanisms of action of different LAB antifungals (Figure 2) [20]. Prevention of mycotoxin production and detoxification are the two main strategies for controlling mycotoxin contamination.

**Table 1.** Antifungal/anti-mycotoxin potential of LAB strains.

Mycotoxin/ (Fungal species)	LAB species for mycotoxin removal	LAB sources	Medium	Percentage reduction of mycotoxins	References
Aflatoxin					
AFB1/ (*Aspergillus flavus, A. parasiticus*)	*L. plantarum*	goat milk	MRS	55%	Escriva et.al (2023) [25]
	*Lactobacillus acidophilus*	probiotic yogurt	MRS-sorbitol agar	46%	Ondiek et.al (2022) [26]
	*Levilactobacillus brevis*	Brazilian artisanal cheeses	MRS	50%	Moller et al. (2021) [27]
AFM1/ (*Aspergillus flavus, A. parasiticus*)	*L. plantarum*	-	MRS	72%	Chaudhary and Patel (2023) [28]
	*Lactococcus lactis ssp. cremoris*	-	MRS	81.4%	Muaz et al. (2021) [29]
Ochratoxin A (OTA)/ (*Aspergillus carbonarius*)	*L. plantarum*	-	MRS	56.8%	Espinosa-Salgado et al. (2022) [30]
	*Lactobacilluskefiranofaciens*	Tibetan kefir grains	MRS	26.9%	Du et al. (2021) [31]
Zearalenone (ZEN)/ (*Fusarium graminearum*)	*Lactobacillus buchneri*	pickles	MRS	72.8%	Gan et al. (2022) [32]
	*Lactobacillus acidophilus*	healthy human urine	PBS	57.4%	Ragoubi et al. (2021) [33]

*Inhibition of fungal growth*

The production of mycotoxins is closely related to the growth status of the fungus, as the onset of mycotoxin production usually occurs at the end of the growth phase of the fungus. Therefore, inhibition of fungal growth is generally considered the most effective strategy to avoid mycotoxin production. When microorganisms co-exist in an environment, the production of growth antagonists and competition between different microorganisms for space and nutrients affect the growth and physiological activities of each other. Living LAB have been found to prevent the growth of fungi through competition for available space and nutrients in an environment in which they co-exist [34]. In apple juice, *Lactobacillus kefiri* M4 prevented the fungus from germinating because when nutrient concentrations were low, LAB preferentially competed for and used nutrients, resulting in insufficient nutrients for fungal growth [35]. Rahayu et al. [36] used *L. plantarum* HL-15 alone or in combination as a fermenting agent for cocoa bean fermentation for inhibiting the growth of *A. niger* and the synthesis of ochratoxin A during fermentation drying. Marie et al. [37] found that *L. plantarum* LO-3 and its CFS inhibited the growth of AF-producing mycobacteria in tomato paste. After inhibiting the growth of the fungus, the production of mycotoxins decreases. LAB can also prevent toxin production by inhibiting the expression of genes involved in aflatoxin synthesis in *Aspergillus flavus* [38]. Furthermore, the metabolites produced during the growth of *L. plantarum* may alter the pH, temperature, moisture, nutrients, and substrate properties of the growth environment. However, completely counteracting the harm caused by mycotoxins by simply inhibiting fungal growth with LAB is difficult. This is because it is possible to reduce the production of mycotoxins only when fungal growth is reduced to a level where mycotoxins cannot be produced due to their retarded growth. So, further research is needed to neutralize mycotoxins. 

*Cell wall adsorption*

**Figure 3.**
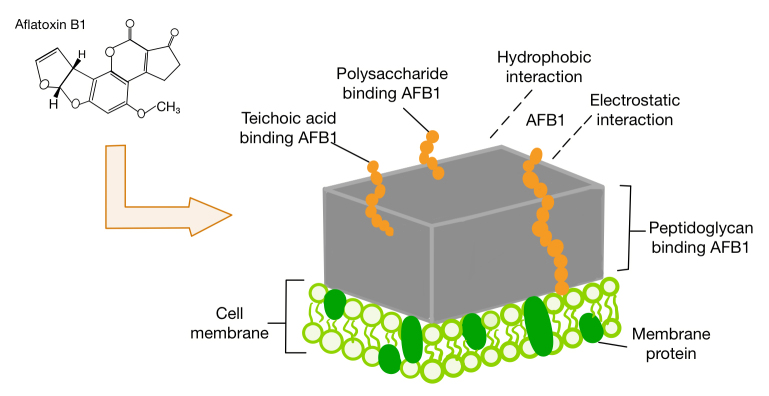
Mechanism of AFs (exemplified by AFB1) adsorption by lactic acid bacteria cell walls. LAB can adsorb AFs via peptidoglycan, polysaccharide, and phosphate in the cell wall, and through hydrophobic and electrostatic interactions.

Aflatoxin removal via cell wall component adsorption by LAB is the most widely studied mechanism (Figure 3) [34]. LAB can adsorb AFs to their cell wall functional groups. Peptidoglycans, carbohydrates, phosphate, or proteins form an intrinsic part of the cell wall of LAB that interacts with the functional groups and binds to the toxin through physical adsorption, ion exchange, and complexation [39, 40]. However, polysaccharides and peptidoglycans are considered the main components of mycotoxin removal by LAB. Differences in the mycotoxin-binding capacity of LAB can be reasonably explained by existing differences in the structure of their cell wall peptidoglycan and the number of available binding sites [41]. Liu et al. [42] observed that AFs had a reversible noncovalent interaction with the cell wall of *L. plantarum* through binding potential, independent of the cellular activity. However, the binding of AFs to the cell wall of *L. plantarum* is affected by factors such as growth medium, bacterial status (alive or dead), incubation temperature, incubation time, and pH value of the medium [34]. Heat and acid treatments significantly affect the structural integrity of bacterial cell wall polysaccharides and peptidoglycans. The cell surface of many *Lactobacillus* strains has been reported to change from strongly hydrophilic to hydrophobic after heat treatment [43], and heat-treated *L. plantarum* was more readily bound to AFs. Ma et al. [44] also reported the highest binding of AFB1 to *L. plantarum* and *Lactobacillus brucei* at pH 2.5, and the lowest binding was observed at pH 8.5. The results indicated that acid treatment increased hydrophobic interactions due to denaturing the cell surface proteins and exposing the more binding area to AFB1.

*Antifungal metabolite action of the fungus*

**Figure 4.**
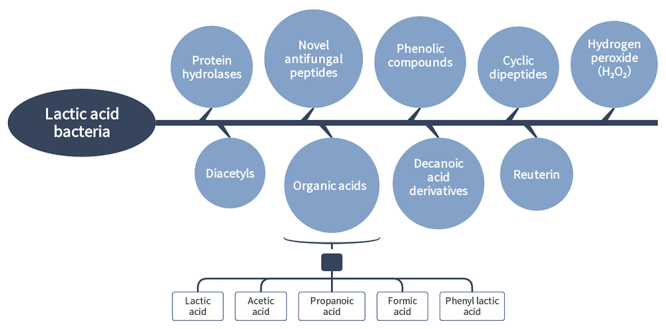
Antifungal metabolites produced by lactic acid bacteria.

LAB degrade AFs by producing several antifungal metabolites, releasing organic acids (such as lactic acid, benzoic and propionic acids, formic acid, butyric acid, caproic and hexanoic acids, and phenyl lactic acid (PLA)), novel antifungal peptides, diacetyl, phenolic compounds, reuterin, bacteriocins, and so forth, during metabolism [45], all of which associated with fungal activity blocking (Figure 4) [46].

*Organic acids*

The main metabolites of Lactobacillus include organic acids, which have a major impact on fungal growth by inhibiting mycelial growth. Organic acids produced by *L. plantarum* metabolism include lactic acid, acetic acid, propionic acid, formic acid, and PLA. Russo et al. [23] revealed that *L. plantarum* had a wide range of antifungal activities due to the high yield of lactic acid. Besides lactic acid, PLA, hydroxy PLA, and indole lactic acid are also found to strongly inhibit AFs production [47]. However, in a recent study, Quattrini et al. [48] reported that acetic acid was the most potent antifungal metabolite of LAB. The mode of action of organic acids is to lower the pH in the environment, causing the inhibition of AFs. The inhibition of organic acids is due to the undissociated form of organic acids. It can diffuse through the cell membrane once internalized into anions and protons. Proton ions contribute to a decrease in internal pH, disrupting proton dynamics and blocking the substrate transport mechanism [49].

*Novel antifungal peptides*

Besides organic acids, inhibitory peptides produced by *L. plantarum* have also been shown to be effective against *A. flavus* and *A. parasiticus* [50]. These antimicrobial peptides are chains of 5-100 amino acids linked together by peptide bonds of natural origin (linked together by peptide bonds). Treatment of the supernatant with protease is a method that can often be used to determine the peptide composition of antifungal compounds. Treatment of *Lactobacillus fermentum* CRL251 supernatant with trypsin, protease K, and pepsin resulted in a 50%, 4%, and 3% reduction in bacteriostatic activity, respectively [51]. Thirty-seven polypeptides were identified from the CFS of *L. plantarum* TE10. Treatment of bread with partial peptides resulted in a fourfold reduction in the microscopic growth and spore formation of *A. flavus* [52]. Muhialdin et al. [53] used Sephadex g-25 (dextran gel) to separate CFS-containing peptides obtained from *L. plantarum* IS10 using size-exclusion chromatography. Fraction 10 showed 60% antifungal activity against *A. flavus* MD3 at a concentration of 0.02 mg/mL peptide. The mechanism of action of antifungal peptides is through binding to carpeted lipid bilayers and penetrating the channels within them, thereby impairing their function.

*Others*

LAB produce a variety of protein hydrolases, including cell wall proteases, peptide transporter proteases, and sufficient intracellular peptidases, which are capable of biodegrading mycotoxins into less toxic and less harmful compounds [5]. Smetankova et al. [54] observed that the mean inhibitory area of *L. plantarum* against *A. flavus* accounted for 10.4% of the total area, among which the antifungal compounds effective against *A. flavus* contained diacetyl. The antifungal mechanism of diacetyl may be due to the induction of ROS (reactive oxygen species) accumulation, which can disrupt the membrane structure and lead to leakage of cellular material and cell death [55]. Gadhoumi et al. [56] found that fermentation of plant extracts with *Lactobacillus* exhibited high antifungal activity, possibly due to an increase in phenolic compounds and flavonoids during the fermentation process. The *Lactobacillus reuteri* strain removed AFB1 at a rate of 26.74% ± 2.17% and was identified as containing a decanoic acid derivative in its antifungal fraction [57]. Kwak et al. [58] isolated 15 proline-containing cyclic dipeptides and one proline-free cyclic dipeptide from *L. plantarum* K10, all exhibiting antifungal activity. The mechanism of action of antibacterial cyclins is primarily through disrupting the structural integrity. They target cell envelope components, leading to membrane cleavage or inhibition of membrane and/or cell wall biosynthesis. In yogurt, reuterin exhibited antifungal effects at a concentration of 1.38 mM [59]. Growth conditions and media can alter the amount of reuterin produced by LAB*.* Reuterin's mechanism of fungal inhibition may be induced by modifying proteins and oxidative stress of sulfhydryl groups in the small molecules [60]. LAB reduce molecular oxygen to hydrogen peroxide, the concentration of which increases dramatically due to the lack of peroxidase activity. High H_2_O_2_ amounts show an oxidative antibacterial effect against various microorganisms [61].

### Factors affecting the degradation of aflatoxins by lactic acid bacteria 

*Types of lactic acid bacteria*

Various species of LAB influence the effectiveness of LAB in degrading AFs. Chlebicz et al. [40] observed that 12 strains of LAB (*Lactobacillus rhamnosus, Lactobacillus casei, Lactobacillus paracasei*, *Lactobacillus reuteri*, and *Lactobacillus brevis*) had variable toxin-binding capacity after 24 h of incubation in PBS (phosphate buffer saline) for 100 μg/L. The final fungal toxin concentrations in the samples ranged from 28.96 to 55.80 μg/mL (44%-71% reduction, average 60%). Martinez et al. [62] observed the degradation of AFM1 in yogurt using three LAB species (*Pediococcus acidilactici, P. pentosaceus,* and *Lactobacillus rhamnosus*), with uptake ranging from 26% to 61%. *Lactobacillus rhamnosus* RC007 showed the highest adsorption rate (61%). Studies have shown [63] that the differences in AFs degradation by different LAB species are mainly due to different cell envelope structures and cell wall components (including the peptidoglycan backbone and protein matrix layer as well as polysaccharides).

*pH*

Gerez et al. [64] demonstrated that the antifungal activity of some *Lactobacillus* strains was lost after neutralization treatment because the acidity of the antifungal metabolites was disrupted. The bioactivity of *L. plantarum* is related to pH. Low pH is the reason why *L. plantarum* has outstanding biological activity against AF-producing strains [23]. Ma et al. [44] also reported the highest binding of AFB1 to *L. plantarum* and *Lactobacillus brucei* at pH 2.5 and the lowest binding at pH 8.5 in phosphate buffers. Another study [47] found that the CFS of *L. plantarum* 55 inhibited the growth of AF-producing fungi (*A. flavus*) by 32% and AFs by 91%, but these inhibitory effects disappeared when the pH of the center was neutralized. As the production of organic acids lowered the pH in the environment, the aforementioned studies suggested that the inhibitory capacity was related to the presence of organic acids. Some studies also demonstrated the pH-dependent antifungal activity of *Lactobacillus* CFS [65].

*Temperature*

Chaudhary et al. [28] found a significant difference in the AFM1-binding capacity of *L. plantarum* S2 at 37 °C and 4 °C (*p* < 0.05). Sokoutifar et al. [66] set three temperature gradients of 4 °C, 21 °C, and 37 °C to investigate the effect of temperature on the degradation of AFM1 in dough by *L. plantarum*. The results showed that *L. plantarum* achieved optimal efficiency in removing AFM1 from dough at 21 °C. The ability of AFM1 to bind to *L. plantarum* was mainly due to the hydrophobic pocket on the surface of the *L. plantarum* cell wall [67]. Heat action may increase the interference of inter and intra-bacterial electrostatic bonds, and subsequently, the cell wall of the probiotic bacteria can form additional bonds with AFM1. The cell wall of *L. plantarum* becomes hydrophobic on increasing the temperature of the medium, suggesting that this feature is responsible for the additional bonding of AFM1 to the surface of the probiotic [68]. So, an appropriate increase in temperature could facilitate the binding of AFM1 by *L. plantarum*.

*Treatment of bacteria*

Abedi et al. [69] inoculated a mixture of *L. plantarum* ATCC 8014 and *Lactobacillus rhamnosus* ATCC 7469 into an AFB1-containing medium in various modes. The different modes were mainly classified as live cells, heat-inactivated cells, and ultrasonically inactivated cells. The final results indicated that the highest AFB1 adsorption was in the order of sonication-inactivated mix (8.042 μg/kg) > heat-treated mix (6.90 μg/kg) > live mix (5.533 μg/kg). This might be because the sonication-inactivated mixes were more stable in binding to AFB1 and less prone to a reversible process to release AFB1. Treatment of AFM1-contaminated milk using live and dead LAB showed that dead cells had better binding capacity than live cells from 4 to 24 h after treatment [70]. This might be due to the increase in available binding sites of dead LAB cells.

### Application of lactic acid bacteria in food and feed

*Lactobacillus* strains have been shown to have the potential for use as antifungal protectants in excess food commodities including dairy products, fruit juices, bakery products, meat, fruit and vegetables, and animal feed, among others.

*Degradation of aflatoxin M1 in dairy products*

Aflatoxin M1 is considered a serious problem in dairy products. In the production and processing of dairy products, raw milk, production process, and storage environment may come into contact with AFM1, and the content of AFM1 may change with temperature, time, and inoculation of LAB. Mousavi Khaneghah et al. [71] found that ripened cheese had less AFM1 than fresh cheese. This may be because the hydrophilic portion of the protein is the binding site for AFM1, and ripened cheese contains less protein than fresh cheese. Moreover, the addition of LAB to cheese production helps to ripen the cheese, and the cell wall components of LAB (peptidoglycan, polysaccharide, etc.) adsorb AFM1, resulting in a reduction in the amount of AFM1 in the cheese [72]. Fermentation of milk into lala (a traditional fermented beverage) and yogurt using LAB has been shown to significantly reduce AFM1 levels. Lala reduced AFM1 by 71.8% after 15 h of incubation at room temperature, and yogurt reduced AFM1 by 73.6% after 4 h of incubation at 45 °C [73]. This may be due to the fact that the pH decreases with incubation time, which may lead to denaturation and coagulation of casein, thus further affecting the adsorption of AFM1 [74]. The inclusion of LAB in dairy products not only helps to enhance the unique flavor and nutritional value of dairy products but also degrades AFM1 in dairy products.

Therefore, the use of LAB to remove AFM1 from milk and its products is the best strategy. For example, Panwar et al. demonstrated [75] the ability of *Lactobacillus* to bind to AFM1 in artificially contaminated skimmed milk. The highest reduction effect of AFM1 was found in the *L. plantarum* LU5, which had the best degradation effect in dairy products (0.5-0.24 μg/kg) [76]. The ability of *L. plantarum* strains to remove AFM1 from fermented milk during storage in a temperature-dependent manner (higher at 21 °C-37 °C) has also been reported [66]. Fakhrabadipour et al. [77] were able to effectively remove 90% of AFM1 after co-incubating a 10^10^ cfu/ml mixture of *Bifidobacterium bifidum* and *Saccharomyces cerevisiae* with AFM1-contaminated skimmed milk for 24 h at 37 °C. 

*Degradation of aflatoxin B1 in bread and silage*

Feed and silage contaminated with AFB1 play an essential role in the production of AFM1 in animals. Lactobacilli are widely used as microbial inoculants in silage, which can improve the aerobic stability of silage. Ma et al. [44] first applied *lactobacilli* in maize silage to test the effect of bacteria in reducing AFB1. *Lactobacillus* species reduced the initial concentration of AFB1 in silage from 30 to 2 µg/kg within 2 h, probably due to the direct binding effect of *Lactobacillus* species. During silage, AFB1 concentrations continued to decrease and reached safe levels after 72 h. Gallo et al. [78] applied a variety of probiotic bacteria to maize silage and showed that the best degradation of AFB1 after 90 days of silage was achieved by *Lactobacillus rhamnosus* LR7 and *L. plantarum* ATCC 8014 silage, with an approximately 50% reduction in AFB1. 

Besides dairy products and silage, the potential of *Lactobacillus* strains to remove mycotoxins has also been used in various other food and feed commodities. For example, the potential of *L. plantarum* strains to remove AFB1 has been used in bread making, where antifungal strains increased the shelf life of bread by three to four days and reduced AFs production in bread by 99.9% compared with the control [79]. Fermented yeast is directly used in the bread recipe to slow down the growth of spoilage microorganisms. Sun et al. [80] found that *L. plantarum* LB-1 significantly improved the textural properties of sourdough and enriched its aroma-volatile compounds, thereby improving the quality and extending the shelf life of whole wheat bread compared with the control group. Luz et al. [81] reported that sourdough bread formulated with whey and additionally fermented with *L. plantarum* CECT 749 or *Lactobacillus bulgaricus* CECT 4005 extended its shelf life by two or four days, respectively. However, despite the antibacterial activity of fermented dough and its aqueous extract, few studies have investigated its antifungal activity.

## Conclusion

Mycotoxin contaminations in food and animal feed are a global concern because of their toxicity, posing risks to human and animal health, in addition to associated economic losses. Therefore, the application and innovation of biodetoxification methods are essential to eliminate mycotoxin contamination and prevent related health problems. Inhibition of mycotoxins using LAB and their active metabolites is a key focus of biodetoxification, which is also a natural tool for preventing fungal growth, extending shelf life, and improving food safety. In recent years, LAB has been used in real-life applications to degrade AFs in bread and silage. Degradation mechanisms include direct inhibition of fungal growth, cell wall adsorption of toxins, and antifungal metabolites (organic acids, antifungal peptides, diacetyl, cyclins, etc.) to degrade toxins. Furthermore, factors such as the diversity of LAB, pH, incubation temperature, and the way the bacteria are treated have been shown to influence the efficiency of LAB in degrading AFs. However, most of these studies have only looked at the changes in the amount of AFs in the presence of LAB by varying these factors, and few have discussed the mechanism of action of these factors in depth. Subsequent studies can delve into how these factors (type of LAB, temperature, pH, treatment of the bacteria, etc.) affect degradation efficiency in terms of (i) inhibition of the growth of AF-producing mycobacteria; (ii) type, quantity, and activity status of the antifungal compounds produced by LAB; (iii) stability of the LAB and AFs binders; and (iv) changes in the structure of the LAB.

Another theory regarding the mechanism of AFs degradation by LAB is that the CFS of LAB downregulates the expression of genes related to AF synthesis. The *aflR* gene is involved in the synthesis of AFs. Ghanbari et al. [82] found that the inhibition of AF production at a concentration of 2 × 10^3^ CFU/mL was due to a significant decrease in the expression level of the *aflR* gene. The CFS of *L. plantarum* MN880325 isolated from ferments, such as batter, reduced AFs yield in peanut kernels by ≥ 97%, and quantitative real-time polymerase chain reaction (qPCR) results showed that its CFS inhibited the *aflR* gene expression by 50% [83]. Recent studies have only confirmed that CFS can downregulate the expression of AF synthesis genes. However, many antifungal compounds exist in the CFS of *Lactobacillus*, and it is yet unclear which components in the CFS downregulate the expression of the *aflR* gene. Further studies can isolate and purify the substances in the CFS, such as organic acids, antifungal peptides, and cyclins, to find the substances that downregulate the expression of the *aflR* gene.

Extensive laboratory evidence suggests that LAB are effective in degrading AFs. Previous studies applied LAB as a biological method to degrade AFs in food or feed and to minimize the health and economic losses caused by toxin contamination. However, in practice, many factors are difficult to strictly control, and the use of LAB in food and feed also requires consideration of aspects such as retaining nutritional value. Pavlek et al. [70] found that after prolonged binding of AFM1 in milk by dead LAB, the trace elements in the milk changed, with the largest deviations in Na, K, and Mg compared with untreated milk. Therefore, further studies are needed on the composition of food and feed, morphology, storage conditions, and the detoxification effect of LAB when multiple combinations of toxins are present together before they can be truly applied in industrial mass production.

### Abbreviations

LAB: lactic acid bacteria; AFs: aflatoxins; AFB1: aflatoxin B1; HCC: hepatic cell carcinoma; IARC: International Agency for Research on Cancer; FAO: Food and Agriculture Organization of the United Nations; *L. plantarum: Lactiplantibacillus plantarum; A. flavus*: *Aspergillus flavus; P. roqueforti: Penicillium roqueforti;* CFS: cell-free supernatant; PLA: phenyl lactic acid; ROS: reactive oxygen species; PBS: phosphate buffer saline; qPCR: quantitative real-time polymerase chain reaction.

## References

[B1] Adebo OA, Molelekoa T, Makhuvele R, Adebiyi JA, Oyedeji AB, Gbashi S, Adefisoye MA, Ogundele OM, Njobeh PB (2021). A review on novel non-thermal food processing techniques for mycotoxin reduction. Int J Food Sci Tech.

[B2] Moretti A, Pascale M, Logrieco AF (2019). Mycotoxin risks under a climate change scenario in Europe. Trends Food Sci Tech.

[B3] Ben Taheur F, Kouidhi B, Al Qurashi YMA, Ben Salah-Abbes J, Chaieb K (2019). Review: Biotechnology of mycotoxins detoxification using microorganisms and enzymes. Toxicon.

[B4] Emmanuel KT, Els V, Bart H, Evelyne D, Els V, Els D (2020). Carry-over of some Fusarium mycotoxins in tissues and eggs of chickens fed experimentally mycotoxin-contaminated diets. Food Chem Toxicol.

[B5] Bangar SP, Sharma N, Kumar M, Ozogul F, Purewal SS, Trif M (2021). Recent developments in applications of lactic acid bacteria against mycotoxin production and fungal contamination. Food Biosci.

[B6] Rushing BR, Selim MI (2019). Aflatoxin B1: A review on metabolism, toxicity, occurrence in food, occupational exposure, and detoxification methods. Food Chem Toxicol.

[B7] Ahmad MM, Qamar F, Saifi M, Abdin MZ (2022). Natural inhibitors: A sustainable way to combat aflatoxins. Front Microbiol.

[B8] Falade TDO, Neya A, Bonkoungou S, Dagno K, Basso A, Senghor AL, Atehnkeng J, Ortega-Beltran A, Bandyopadhyay R (2022). Aflatoxin Contamination of Maize, Groundnut, and Sorghum Grown in Burkina Faso, Mali, and Niger and Aflatoxin Exposure Assessment. Toxins (Basel).

[B9] Hamad GM, Mehany T, Simal-Gandara J, Abou-Alella S, Esua OJ, Abdel-Wahhab MA, Hafez EE (2023). A review of recent innovative strategies for controlling mycotoxins in foods. Food Control.

[B10] Ajmal M, Bedale W, Akram A, Yu JH (2022). Comprehensive Review of Aflatoxin Contamination, Impact on Health and Food Security, and Management Strategies in Pakistan. Toxins (Basel).

[B11] Kumar P, Mahato DK, Kamle M, Mohanta TK, Kang SG (2017). Aflatoxins: A Global Concern for Food Safety, Human Health and Their Management. Front Microbiol.

[B12] Benkerroum N (2020). Chronic and Acute Toxicities of Aflatoxins: Mechanisms of Action. Int J Environ Res Public Health.

[B13] Ostry V, Malir F, Toman J, Grosse Y (2017). Mycotoxins as human carcinogens-the IARC Monographs classification. Mycotoxin Res.

[B14] Coskun E, Faruga P, Vartanian V, Erdem O, Egner PA, Groopman JD, Lloyd RS, Dizdaroglu M (2019). Aflatoxin-Guanine DNA Adducts and Oxidatively Induced DNA Damage in Aflatoxin-Treated Mice in Vivo as Measured by Liquid Chromatography-Tandem Mass Spectrometry with Isotope Dilution. Chem Res Toxicol.

[B15] Patil H, Shah NG, Hajare SN, Gautam S, Kumar G (2019). Combination of microwave and gamma irradiation for reduction of aflatoxin B-1 and microbiological contamination in peanuts (Arachis hypogaea L.). World Mycotoxin J.

[B16] Ben Amara A, Mehrez A, Ragoubi C, Romero-Gonzalez R, Frenich AG, Landoulsi A, Maatouk I (2022). Fungal mycotoxins reduction by gamma irradiation in naturally contaminated sorghum. J Food Process Pres.

[B17] Esua OJ, Sun DW, Cheng JH, Wang HF, Chen C (2022). Hybridising plasma functionalized water and ultrasound pretreatment for enzymatic protein hydrolysis of Larimichthys polyactis: Parametric screening and optimization. Food Chem.

[B18] Porto YD, Trombete FM, Freitas-Silva O, de Castro IM, Direito GM, Ascheri JLR (2019). Gaseous Ozonation to Reduce Aflatoxins Levels and Microbial Contamination in Corn Grits. Microorganisms.

[B19] Grenier B, Loureiro-Bracarense A-P, Leslie JF, Oswald IP (2014). Physical and Chemical Methods for Mycotoxin Decontamination in Maize. Mycotoxin Reduction in Grain Chains.

[B20] Ren XF, Zhang Q, Zhang W, Mao J, Li PW (2020). Control of Aflatoxigenic Molds by Antagonistic Microorganisms: Inhibitory Behaviors, Bioactive Compounds, Related Mechanisms, and Influencing Factors. Toxins (Basel).

[B21] Ahlberg SH, Joutsjoki V, Korhonen HJ (2015). Potential of lactic acid bacteria in aflatoxin risk mitigation. Int J Food Microbiol.

[B22] Shehata MG, Badr AN, El Sohaimy SA, Asker D, Awad TS (2019). Characterization of antifungal metabolites produced by novel lactic acid bacterium and their potential application as food biopreservatives. Ann Agr Sci-Cairo.

[B23] Russo P, Arena MP, Fiocco D, Capozzi V, Drider D, Spano G (2017). Lactobacillus plantarum with broad antifungal activity: A promising approach to increase safety and shelf-life of cereal-based products. Int J Food Microbiol.

[B24] Nazareth TM, Luz C, Torrijos R, Quiles JM, Luciano FB, Manes J, Meca G (2019). Potential Application of Lactic Acid Bacteria to Reduce Aflatoxin B1 and Fumonisin B1 Occurrence on Corn Kernels and Corn Ears. Toxins (Basel).

[B25] Escriva L, Calpe J, Lafuente C, Moreno A, Musto L, Meca G, Luz C (2023). Aflatoxin B1 and ochratoxin A reduction by Lactobacillus spp. during bread making. J Sci Food Agric.

[B26] Ondiek W, Wang Y, Sun L, Zhou L, On SL, Zheng H, Ravi G (2022). Removal of aflatoxin b1 and t-2 toxin by bacteria isolated from commercially available probiotic dairy foods. Food Sci Technol Int.

[B27] Moller COA, Freire L, Rosim RE, Margalho LP, Balthazar CF, Franco LT, Sant'Ana AS, Corassin CH, Rattray FP, de Oliveira CAF (2021). Effect of Lactic Acid Bacteria Strains on the Growth and Aflatoxin Production Potential of Aspergillus parasiticus, and Their Ability to Bind Aflatoxin B1, Ochratoxin A, and Zearalenone in vitro. Front Microbiol.

[B28] Chaudhary HJ, Patel AR (2022). Removal of aflatoxin M1 from milk and aqueous medium by indigenously isolated strains of W. confusa H1 and L. plantarum S2. Food Biosci.

[B29] Muaz K, Riaz M, Rosim RE, Akhtar S, Corassin CH, Goncalves BL, Oliveira CAF (2021). In vitro ability of nonviable cells of lactic acid bacteria strains in combination with sorbitan monostearate to bind to aflatoxin M1 in skimmed milk. LWT-Food Sci Technol.

[B30] Espinosa-Salgado R, Tamayo-Galvan V, Perraud-Gaime I, Rodriguez-Serrano GM, Gonzalez-Robles RO, Durand N, Champion-Martinez EI, Saucedo-Castaneda G (2022). Polyols Induce the Production of Antifungal Compounds by *Lactobacillus plantarum*. Curr Microbiol.

[B31] Du G, Liu L, Guo Q, Cui Y, Chen H, Yuan Y, Wang Z, Gao Z, Sheng Q, Yue T (2021). Microbial community diversity associated with Tibetan kefir grains and its detoxification of Ochratoxin A during fermentation. Food Microbiol.

[B32] Gan M, Hu J, Wan K, Liu X, Chen P, Zeng R, Wang F, Zhao Y (2022). Isolation and Characterization of Lactobacillus paracasei 85 and Lactobacillus buchneri 93 to Absorb and Biotransform Zearalenone. Toxics.

[B33] Ragoubi C, Quintieri L, Greco D, Mehrez A, Maatouk I, D'Ascanio V, Landoulsi A, Avantaggiato G (2021). Mycotoxin Removal by Lactobacillus spp. and Their Application in Animal Liquid Feed. Toxins (Basel).

[B34] Sadiq FA, Yan BW, Tian FW, Zhao JX, Zhang H, Chen W (2019). Lactic Acid Bacteria as Antifungal and Anti-Mycotoxigenic Agents: A Comprehensive Review. Compr Rev Food Sci Food Saf.

[B35] Chen H, Ju HM, Wang YW, Du GA, Yan XH, Cui YY, Yuan YH, Yue TL (2021). Antifungal activity and mode of action of lactic acid bacteria isolated from kefir against Penicillium expansum. Food Control.

[B36] Rahayu ES, Triyadi R, Khusna RNB, Djaafar TF, Utami T, Marwati T, Hatmi RU (2021). Indigenous Yeast, Lactic Acid Bacteria, and Acetic Acid Bacteria from Cocoa Bean Fermentation in Indonesia Can Inhibit Fungal-Growth-Producing Mycotoxins. Fermentation-Basel.

[B37] Marie KP, Francois ZN, Marius FKE, Ciobotaru O, Matei F, Cornea CP, Israel-Roming F (2018). Antifungal Activity of Lactic Acid Bacteria Isolated from Peanuts, Gari, and Orange Fruit Juice against Food Aflatoxigenic Molds. Food Biotechnol.

[B38] Mendonca CMN, Oliveira RC, Pizauro LJL, Pereira WA, Abboud K, Almeida S, Watanabe IS, Varani AM, Dominguez JM, Correa B, Venema K, Azevedo POS, Oliveira RPS (2023). Tracking new insights into antifungal and anti-mycotoxigenic properties of a biofilm forming Pediococcus pentosaceus strain isolated from grain silage. Int J Food Microbiol.

[B39] Asurmendi P, Gerbaldo G, Pascual L, Barberis L (2020). Lactic acid bacteria with promising AFB1 binding properties as an alternative strategy to mitigate contamination on brewers' grains. J Environ Sci Health B.

[B40] Chlebicz A, Slizewska K (2020). In Vitro Detoxification of Aflatoxin B1, Deoxynivalenol, Fumonisins, T-2 Toxin and Zearalenone by Probiotic Bacteria from Genus Lactobacillus and Saccharomyces cerevisiae Yeast. Probiotics Antimicrob Proteins.

[B41] Lili Z, Junyan W, Hongfei Z, Baoqing Z, Bolin Z (2018). Detoxification of cancerogenic compounds by lactic acid bacteria strains. Crit Rev Food Sci Nutr.

[B42] Liu A, Zheng Y, Liu L, Chen S, He L, Ao X, Yang Y, Liu S (2020). Decontamination of Aflatoxins by Lactic Acid Bacteria. Curr Microbiol.

[B43] Piotrowska M (2014). The adsorption of ochratoxin A by lactobacillus species. Toxins (Basel).

[B44] Ma ZX, Amaro FX, Romero JJ, Pereira OG, Jeong KC, Adesogan AT (2017). The capacity of silage inoculant bacteria to bind aflatoxin B1 in vitro and in artificially contaminated corn silage. J Dairy Sci.

[B45] Ruggirello M, Nucera D, Cannoni M, Peraino A, Rosso F, Fontana M, Cocolin L, Dolci P (2019). Antifungal activity of yeasts and lactic acid bacteria isolated from cocoa bean fermentations. Food Res Int.

[B46] Liu A, Xu R, Zhang S, Wang Y, Hu B, Ao X, Li Q, Li J, Hu K, Yang Y, Liu S (2022). Antifungal Mechanisms and Application of Lactic Acid Bacteria in Bakery Products: A Review. Front Microbiol.

[B47] Guimaraes A, Santiago A, Teixeira JA, Venancio A, Abrunhosa L (2018). Anti-aflatoxigenic effect of organic acids produced by Lactobacillus plantarum. Int J Food Microbiol.

[B48] Quattrini M, Liang N, Fortina MG, Xiang S, Curtis JM, Ganzle M (2019). Exploiting synergies of sourdough and antifungal organic acids to delay fungal spoilage of bread. Int J Food Microbiol.

[B49] Saelim K, Jampaphaeng K, Maneerat S (2017). Functional properties of Lactobacillus plantarum S0/7 isolated fermented stinky bean (Sa Taw Dong) and its use as a starter culture. J Funct Foods.

[B50] Luz C, Saladino R, Luciano FB, Manes J, Meca G (2017). In vitro antifungal activity of bioactive peptides produced by Lactobacillus plantarum against Aspergillus parasiticus and Penicillium expansum. LWT-Food Sci Technol.

[B51] Nasrollahzadeh A, Mokhtari S, Khomeiri M, Saris PEJ (2022). Antifungal Preservation of Food by Lactic Acid Bacteria. Foods.

[B52] Muhialdin BJ, Algboory HL, Kadum H, Mohammed NK, Saari N, Hassan Z, Hussin ASM (2020). Antifungal activity determination for the peptides generated by Lactobacillus plantarum TE10 against Aspergillus flavus in maize seeds. Food Control.

[B53] Muhialdin BJ, Hassan Z, Saari N (2018). In vitro antifungal activity of lactic acid bacteria low molecular peptides against spoilage fungi of bakery products. Ann Microbiol.

[B54] Smetankova J, Hladikova Z, Zimanova M, Greif G, Greifova M (2014). Lactobacilli Isolated from Lump Sheep's Cheeses and their Antimicrobial Properties. Czech J Food Sci.

[B55] Shi C, Knochel S (2021). Susceptibility of dairy associated molds towards microbial metabolites with focus on the response to diacetyl. Food Control.

[B56] Gadhoumi H, Hayouni ELA, Martinez-Rojas E, Yeddes W, Tounsi MS (2022). Biochemical composition, antimicrobial and antifungal activities assessment of the fermented medicinal plants extract using lactic acid bacteria. Arch Microbiol.

[B57] Sadeghi A, Ebrahimi M, Mortazavi SA, Abedfar A (2019). Application of the selected antifungal LAB isolate as a protective starter culture in pan whole-wheat sourdough bread. Food Control.

[B58] Kwak MK, Liu R, Kang SO (2018). Antimicrobial activity of cyclic dipeptides produced by Lactobacillus plantarum LBP-K10 against multidrug-resistant bacteria, pathogenic fungi, and influenza A virus. Food Control.

[B59] Vimont A, Fernandez B, Ahmed G, Fortin HP, Fliss I (2019). Quantitative antifungal activity of reuterin against food isolates of yeasts and moulds and its potential application in yogurt. Int J Food Microbiol.

[B60] Singh VP (2018). Recent approaches in food bio-preservation - a review. Open Vet J.

[B61] Alharbi NK, Alsaloom AN (2021). Characterization of Lactic Bacteria Isolated from Raw Milk and Their Antibacterial Activity against Bacteria as the Cause of Clinical Bovine Mastitis. J Food Quality.

[B62] Martinez MP, Magnoli AP, Gonzalez Pereyra ML, Cavaglieri L (2019). Probiotic bacteria and yeasts adsorb aflatoxin M1 in milk and degrade it to less toxic AFM1-metabolites. Toxicon.

[B63] Danial EN, Lamfon MY, Alghamdi LA, Alamri AM, Alghamdi MS, Alghamdi SA (2021). Removal of aflatoxin G1 using lactic acid bacteria. J Food Process Pres.

[B64] Gerez CL, Torres MJ, de Valdez GF, Rollan G (2013). Control of spoilage fungi by lactic acid bacteria. Biol Control.

[B65] Gajbhiye M, Kapadnis B (2021). Lactococcus lactis subsp. cremoris of Plant Origin Produces Antifungal Cyclo-(Leu-Pro) and Tetradecanoic Acid. Indian J Microbiol.

[B66] Sokoutifar R, Razavilar V, Anvar AA, Shoeiby S (2018). Degraded aflatoxin M1 in artificially contaminated fermented milk using Lactobacillus acidophilus and Lactobacillus plantarum affected by some bio-physical factors. J Food Safety.

[B67] Haskard CA, El-Nezami HS, Kankaanpaa PE, Salminen S, Ahokas JT (2001). Surface Binding of Aflatoxin B1 by Lactic Acid Bacteria. Appl Environ Microbiol.

[B68] Jebali R, Abbes S, Salah-Abbes JB, Younes RB, Haous Z, Oueslati R (2015). Ability of Lactobacillus plantarum MON03 to mitigate aflatoxins (B1 and M1) immunotoxicities in mice. J Immunotoxicol.

[B69] Abedi E, Pourmohammadi K, Mousavifard M, Sayadi M (2022). Comparison between surface hydrophobicity of heated and thermosonicated cells to detoxify aflatoxin B1 by co-culture Lactobacillus plantarum and Lactobacillus rhamnosus in sourdough: Modeling studies. LWT-Food Sci Technol.

[B70] Pavlek Z, Bosnir J, Kuharic Z, Racz A, Jurak I, Lasic D, Markov K, Jakopovic Z, Frece J (2022). The Influence of Binding of Selected Mycotoxin Deactivators and Aflatoxin M1 on the Content of Selected Micronutrients in Milk. Processes.

[B71] Mousavi Khaneghah A, Moosavi M, Omar SS, Oliveira CAF, Karimi-Dehkordi M, Fakhri Y, Huseyn E, Nematollahi A, Farahani M, Sant'Ana AS (2021). The prevalence and concentration of aflatoxin M1 among different types of cheeses: A global systematic review, meta-analysis, and meta-regression. Food Control.

[B72] Mohammadi S, Keshavarzi M, Kazemi A, Rahmdel S, Nouri M, Rastegar A, Ghaffarian‐Bahraman A (2022). Aflatoxin‐M1 contamination in cheese of six countries in the West Asia region: A systematic review and meta‐analysis. Int J Dairy Technol.

[B73] Kuboka MM, Imungi JK, Njue L, Mutua F, Grace D, Lindahl JF (2019). Occurrence of aflatoxin M1 in raw milk traded in peri-urban Nairobi, and the effect of boiling and fermentation. Infect Ecol Epidemiol.

[B74] Hattimare D, Shakya S, Patyal A, Chandrakar C, Kumar A (2022). Occurrence and exposure assessment of Aflatoxin M1 in milk and milk products in India. J Food Sci Technol.

[B75] Panwar R, Kumar N, Kashyap V, Ram C, Kapila R (2019). Aflatoxin M1 Detoxification Ability of Probiotic Lactobacilli of Indian Origin in In vitro Digestion Model. Probiotics Antimicrob Proteins.

[B76] Hashemi SMB, Gholamhosseinpour A (2019). Fermentation of table cream by Lactobacillus plantarum strains: effect on fungal growth, aflatoxin M1 and ochratoxin A. Int J Food Sci Technol.

[B77] Fakhrabadipour M, Khajehrahimi AE, Haghdoost NS, Anvar SA, Tala M (2023). Efficiency of Bifidobacterium bifidum and Saccharomyces cerevisiae for detoxification of aflatoxin M1 in skim milk. Int J Dairy Technol.

[B78] Gallo A, Fancello F, Ghilardelli F, Zara S, Froldi F, Spanghero M (2021). Effects of several lactic acid bacteria inoculants on fermentation and mycotoxins in corn silage. Anim Feed Sci Tech.

[B79] Saladino F, Luz C, Manyes L, Fernandez-Franzon M, Meca G (2016). In vitro antifungal activity of lactic acid bacteria against mycotoxigenic fungi and their application in loaf bread shelf life improvement. Food Control.

[B80] Sun L, Li XF, Zhang YY, Yang WJ, Ma GX, Ma N, Hu QH, Pei F (2020). A novel lactic acid bacterium for improving the quality and shelf life of whole wheat bread. Food Control.

[B81] Luz C, D'Opazo V, Manes J, Meca G (2019). Antifungal activity and shelf life extension of loaf bread produced with sourdough fermented by Lactobacillus strains. J Food Process Pres.

[B82] Ghanbari R, Aghaee EM, Rezaie S, Khaniki GJ, Alimohammadi M, Soleimani M, Noorbakhsh F (2018). The inhibitory effect of lactic acid bacteria on aflatoxin production and expression of aflR gene in Aspergillus parasiticus. J Food Safety.

[B83] Pradhan S, Ananthanarayan L, Prasad K, Bhatnagar-Mathur P (2021). Anti-fungal activity of lactic acid bacterial isolates against aflatoxigenic fungi inoculated on peanut kernels. LWT-Food Sci Technol.

